# Molecular Cloning and Characterization of an Acetylcholinesterase cDNA in the Brown Planthopper, *Nilaparvata lugens*

**DOI:** 10.1673/031.010.10201

**Published:** 2010-07-11

**Authors:** Zhifan Yang, Jun Chen, Yongqin Chen, Sijing Jiang

**Affiliations:** ^1^ College of Life Sciences, Hubei University, Wuhan 430062, China; ^2^ College of Chemical Engineering and Technology, Wuhan University of Science and Technology, Wuhan 430081, China

**Keywords:** phylogenetic analysis, insecticide resistance, gene mutation

## Abstract

A full cDNA encoding an acetylcholinesterase (AChE, EC 3.1.1.7) was cloned and characterized from the brown planthopper, *Nilaparvata lugens* Stål (Hemiptera: Delphacidae). The complete cDNA (2467 bp) contains a 1938-bp open reading frame encoding 646 amino acid residues. The amino acid sequence of the AChE deduced from the cDNA consists of 30 residues for a putative signal peptide and 616 residues for the mature protein with a predicted molecular weight of 69,418. The three residues (Ser242, Glu371, and His485) that putatively form the catalytic triad and the six Cys that form intra-subunit disulfide bonds are completely conserved, and 10 out of the 14 aromatic residues lining the active site gorge of the AChE are also conserved. Northern blot analysis of poly(A)^+^ RNA showed an approximately 2.6-kb transcript, and Southern blot analysis revealed there likely was just a single copy of this gene in *N. lugens*. The deduced protein sequence is most similar to AChE of *Nephotettix cincticeps* with 83% amino acid identity. Phylogenetic analysis constructed with 45 AChEs from 30 species showed that the deduced *N. lugens* AChE formed a cluster with the other 8 insect AChE2s. Additionally, the hypervariable region and amino acids specific to insect AChE2 also existed in the AChE of *N. lugens*. The results revealed that the AChE cDNA cloned in this work belongs to insect AChE2 subgroup, which is orthologous to *Drosophila* AChE. Comparison of the AChEs between the susceptible and resistant strains revealed a point mutation, Gly185Ser, is likely responsible for the insensitivity of the AChE to methamidopho in the resistant strain.

## Introduction

Acetylcholinesterase (AChE) catalyses the hydrolysis of the neurotransmitter, acetylcholine, thereby stopping transmission of nerve impulses at synapses of cholinergic neurons in the central and peripheral nervous systems in both vertebrates and invertebrates (Taylor 1991). Consequently, inhibition of AChE leads to paralysis and death. In addition, AChEs are expressed at other sites in animals, where they may act as regulators involved in cell growth and adhesion, probably unrelated to their catalytic properties (Soreq and Seidman 2001). In insects, AChE is a target of organophosphorus and carbamate compounds, which remain widely used pesticides around the world (Harel et al. 2000).

Since the first cloning of an insect AChE gene (*Ace*) from *Drosophila melanogaster* (Hall and Spierer 1986), 602 AChE sequences from Arthropoda (551 in Hexapoda and 51 in Ixodidae) have been registered with databases (http://www.uniprot.org/uniprot/?by=taxonomy&query=prosite+PS00941#35237, 2759, 33208, 119089, 6656, 6939, 6960, 33340, 33342, 7524). Biochemical characterizations of AChE have been carried out in more than 20 insect species (Gao et al. 1998). Gene structures of AChEs from the economic and medical insect species have been characterized in detail, including *Anopheles stephensi* (Hall and Malcolm 1991), *Aedes aegypti* (Anthony et al. 1995; Mori et al. 2007), *Leptinotarsa decemlineata* (Zhu and Clark 1995), *Musca domestica* (Huang et al. 1997), *Nephotettix cincticeps* (Tomita et al. 2000), *Schizaphis graminum* (Gao et al. 2002), *Nippostrongylus brasiliensis* (Hussein et al. 2002), *Aphis gossypii* (Li and Han 2004; Toda et al. 2008), *Culex tritaeniorhynchus* (Nabeshima et al. 2004), *Blattella germanica* (Mizuno et al. 2007), and *Alphitobius diaperinus* (Kozaki et al. 2008). These studies helped in revealing the molecular structure of the insect AChEs and the mechanism of insecticide-resistance in these important insect pests.

The brown planthopper *Nilaparvata lugens* Stål (Hemiptera: Delphacidae), is one of the most important agricultural pests in rice planting areas. It is a rice specialist feeder that often causes serious loss of rice yield by sucking sap from the phloem and by transmitting the stunt virus disease (Rubia-Sanchez et al. 1999). Insecticides are commonly used to control *N. lugens* in field, but this often causes insecticide-resistance and resurgence of the insect pest (Sujatha and Regupathy 2003). An altered AChE has been verified in *N. lugens* as a common mechanism of resistance to organophosphorus and carbamates (Yoo et al. 2002). However, the structure of the AChE gene from *N. lugens* remains to be elucidated. Cloning of the AChE cDNA is expected to lay a foundation for understanding the molecular properties of the AChE from *N. lugens*.

In this paper, data is presented on cDNA cloning and characterization, as well as the comparison of an AChE from methamidophosensitive and -insensitive *N. lugens* strains. The following aspects are reported: (1) the AChE cDNA nucleotide sequence and its deduced amino acid sequence; (2) characteristics of the cDNA-deduced AChE; (3) phylogenetic analysis of this AChE relative to those from other animals; (4) the AChE transcript size and expression level, as well as the gene copy in the genome; and (5) detection of resistance-associated point mutations of methamidopho-insensitive acetylcholinesterase in the resistant strain.

## Materials and Methods

### Experimental insects

The clone of the susceptible *N. lugens* was mass reared on plants of Taichung Native 1 at 25 ± 2° C, 80% relative humidity, under the photoperiod of 16:8 L:D. Adult insects were collected for genomic DNA isolation. Fourth instar larvae and adults were used for RNA isolation.

Resistant *N. lugens* was collected originally from Xianning district, Hubei Province, China, where usage of methamidopho to control this pest was widespread. After screening with methamidopho (50% emulsion, technical grade, Hubei Sanongda Pesticide Co. Ltd.), a single colony was selected to construct field-resistant clones. The Median Lethal Dose, 50%, (LD_50_) of methamidopho to the resistant strain was 0.150 !g (volume converted to mass) per fourth instar larva, while the dose to the susceptible strain was 0.006 !g per larva. Thus the resistant strain showed a moderate resistance level to methamidopho (resistance ratio: 25). The resistant strain for RNA isolation was reared as described above.

### Cloning of AChE cDNA fragments

Total RNA was isolated from fourth instar larvae by TRIzol reagent (Invitrogen, www.invitrogen.com). poly(A)^+^ RNA was separated from total RNA (1 mg) using oligo (dT) coupled with paramagnetic beads (Promega, www.promega.com). The first and second strand cDNA was synthesized according to standard protocols (Sambrook et al. 1989). The double-stranded cDNA was purified and dissolved in Tris-EDTA buffer solution (10mM Tris-HCl, 1.0mM EDTA, pH 8.0).

**Table 1.**
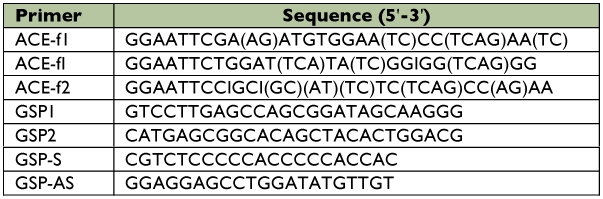
Primers used for RT-PCR and RACE reactions.

A 278-bp homologous AChE cDNA fragment was generated using semi-nested PCR and degenerate primers (ACE-f1, ACE-f_I_, and ACE-f_2_) (Table 1), as described by Zhu et al. (2000). The 278-bp cDNA fragment was used as template to design anti-sense and sense gene specific primers (GSP1 and GSP2) for 5′ and 3′ RACE reactions. The sequences of primers are listed in Table 1.

ACE-f1, ACE-f_I_, and ACE-f2 indicate forward primer, forward inner primer and reverse primer, respectively. GSP1 and GSP2 indicate the gene specific primers for 5′ and 3′ RACE, respectively. GSP-S and GSP-AS indicate the gene specific primers for amplification of the inner cDNA fragment containing the complete coding region of the acetylcholinesterase.

### 5′ and 3′ rapid amplification of cDNA ends (RACE)

The 5′ and 3′ RACE reactions were carried out according to the instruction manual of the SMART RACE cDNA Amplification Kit (BD Bioscience Clontech Company, www.clontech.com ). cDNAs were synthesized by using primers, 5′-CDS, 3′-CDS and SMART II A Oligo provided by the kit. The 5′ end of cDNA was amplified by using GSP1 and UMP, and the 3′-end of cDNA was amplified by using GSP2 and UMP. Touchdown amplification profiles were used as follows: 94° C for 30 s, 72° C for 3 min for

5 cycles, then 94° C for 30 s, 70° C for 30 s and 72° C for 3 min for 5 cycles, then continued at 94° C for 30 s, 68° C for 30 s and 72° C for 3 min for the remaining 28 cycles. Amplified fragments were routinely cloned into pGEMT vectors (Promega) and sequenced using M13 and M13(-) universal primers at both ends. More than 4 independent clones of each, the 5′ and 3′ ends of cDNAs, were sequenced to eliminate possible PCR mutations.

### Sequencing and computer-assisted analysis of AChE cDNA

Molecular mass and isoelectric point were predicted by Compute pI / Mw tool (http://us.expasy.org/tools/pi_tool.html). Signal peptide was predicted by SignalP 3.0 Server (http://www.cbs.dtu.dk/services/SignalP). A molecular phylogenetic tree was con-structed with PAUP 4.0 software using the bootstrap N-J tree option (*n* of bootstrap trials = 1000). The tree was viewed by using Tree View (v. 1.6.6). Potential N-linked glycosylation sites were predicted by using NetNglyc program (Nielsen et al. 1997)

### Southern and Northern blot analysis

A DNA fragment (1056-bp, 36-1092 in the full cDNA) was generated by digestion of the 5′ end cDNA with *Xho* I and *Nhe* I (Promega). The DNA fragment was used as a probe for Southern and Northern blot analyses. The probe was labeled by random primer using “ [^32^P] dCTP (Perkin Elmer Life Sciences, www.perkinelmer.com).

The poly(A)^+^ RNA was separated from total RNA of adult *N. lugens* and fourth instar larvae and analyzed by electrophoresis on 1.5% denaturing, formaldehyde agarose gels (3 µg each lane). An outer lane containing RNA markers was excised from the gel prior to blotting, stained with ethidium bromide and used for size estimations. The RNA gel was blotted onto a Hybond-N^+^ nylon membrane (Amersham, USA) that was then denatured by alkali and subsequently baked at 80° C for 2 h. The filter was prehybridized for 6 h at 65° C, then hybridized overnight at 65° C, washed in I×SSC, 0.2% (weight/volume) SDS at 65° C for 15 min, and then in 0.5×SSC, 0.1% (weight/volume) SDS at 65° C for another 15 min, then exposed to X-ray films (FUJI Film, www.fujifilm.com) for one week at -80° C.

Genomic DNA was isolated from adult *N. lugens* according to Sambrook et al. (1989). Aliquots containing 15 µg genomic DNA were digested with *Eco* RI, *Eco* RV, *Hind* III, and *Dra* I (Promega), and the resulting fragments were separated by electrophoresis in a 1.5% agarose gel, then transferred to an NC membrane (Amersham) that was then baked at 80° C for 2 h. DNA markers were disposed as described in the case of the Northern blot procedure. The filter was prehybridized for 6 h at 65° C, then hybridized overnight at 65° C with the labeled probe. The membrane was washed, and autoradiography was performed as above.

### Detection of mutations in AChE possibly associated with methamidopho resistance

To compare the nucleotide sequences of AChE cDNA between the resistant strain and the susceptible strain, and to find the mutations potentially involved in methamidopho resistance, 5 insect larvae from each strain were separately isolated for total RNA by using an RNA isolation kit (Takara, www.takara-bio.com). The cDNA retro transcribed from total RNA was used as template, and an inner AChE2 cDNA fragment was amplified by long distance-PCR with gene specific primers, GSP-S and GSP-AS (Table 1). The following amplification profiles were used: 94° C for 2 min; 94° C for 30 s, 55° C for 30 s, and 72° C for 3 min for 35 cycles; then 72° C for 10 min. All amplification reactions were performed in a PE-9700 PCR machine (Perkin Elmer). Amplified fragments were cloned and sequenced.

## Results

### Cloning and characterization of AChE cDNA from *N. Lugens*

**Figure 1.**
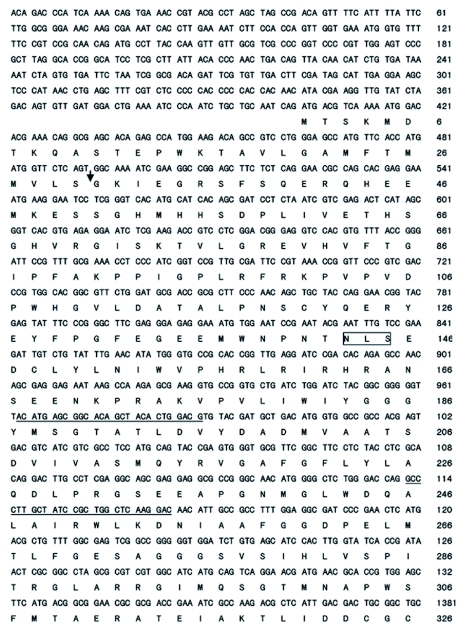
Nucleotide and deduced amino acid sequences of AChE cDNA from *Nilapavarta lugens*. The signal peptide cleavage site is marked by a vertical arrow. Four potential N-linked glycosylation sites are boxed. The stop codon at the end of the coding region is marked with an asterisk (*). A putative polyadenylation signal (AATAAA) in the 3′-untranslated sequence is underlined with dots. The sequences of GSPs for 5′ and 3′ RACE are underlined. This sequence was deposited in the GenBank (accession number: AJ8S2420). High quality figures are available online.

From the PCR on *N. lugens* cDNA using degenerate primers, a 278-bp cDNA fragment with deduced amino acid sequences that matched AChEs in GenBank was generated. A 2467-bp full-length cDNA sequence was obtained by RACE reactions based on the 278-bp cDNA fragment. The full sequence consisted of a 5′ untranslated region (UTR) of 403 bp, an open reading frame of 1938 bp, and a 3′ UTR of 123 bp including a poly(A) tail of 29 bp. The 3′ UTR possessed a typical polyadenylation signal (AATAAA) 14 bp upstream of the poly(A) tail (Figure 1).

A putative preproenzyme of 646 amino acid residues was encoded by the open reading frame of the cloned AChE cDNA. The predicted preproenzyme comprised a signal peptide of 30 amino acid residues at the N-terminal predicted by SignalP 3.0 Server and a mature enzyme of 616 amino acids (Figure 1). The predicted molecular mass and isoelectric point of the mature enzyme were 69418.57 and 5.21, respectively, which are close to those of AChE from *N. cincticeps* (Mw / pI: 73764.15 / 5.30; Ac: AF145235-1) and *A. gossypii* (Mw / pI: 70541.51 / 4.98; Ac: AF502081-1). There were four potential N-glycosylation sites in the amino acid sequence of the deduced mature AChE (Asn-X-Ser or Asn-X-Thr) (von Heijne 1987). The four sites were located at 113–115 (i.e. Asn-Leu-Ser), 407–409 (i.e. Asn-Met-Thr), 498–500 (i.e. Asn-Met-Ser), and 605–607 (i.e. Asn-Met-Thr) (Figure 1).

### Characterization of the cDNA-deduced AChE

**Figure 2.**
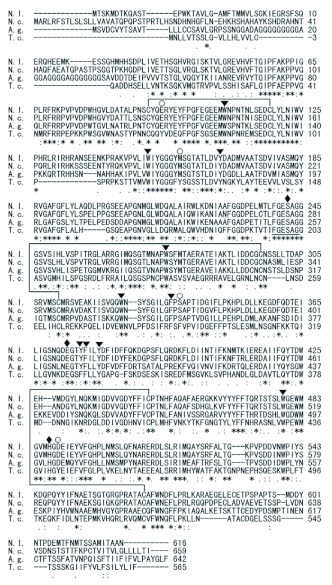
Alignment of *Nilavaparta lugens* (N.l.), *Nephotettix cincticeps* (N.c), *Aphis gossypii* (A.g.) and *Torpedo californica* (T.c.) AChE sequences. Numbering of the amino acid sequences is from the N-terminus of mature proteins. Identical amino acids are indicated by asterisks (*) and conservative substitutions by dots. The residues forming catalytic triads are depicted with diamonds. Cysteine residues involved in intrachain disulfide bonds are connected by lines. The positions of conserved aromatic residues lining the active site gorge are marked with triangles. The positions of non-aromatic residues that possibly substitute the aromatic residues lining the active site gorge are marked with cycles. The Cholinesterase signature sequence is underlined. High quality figures are available online.

For the primary structure of the protein, *N. lugens* exhibits all the major conserved features revealed by AChE of *Torpedo californica* (Ac: P04058) (Sussman et al. 1991) (Figure 2). The structure features are as follows (AChE amino acids are numbered from the start of the mature proteins and the corresponding amino acid residues for *T. californica* are listed in parentheses for reference): (1) conserved active site triad: S242 (200), E371 (327), and H485 (440); (2) a choline binding site: W108 (84); (3) three pairs of cysteines putatively forming intrachain disulfide bonds (C91 (67)-C118 (94), C296 (254)-C311 (265), and C447 (402)-C563 (521)); (4) the sequence FGESAG, flanking S242 (200), conserved in all cholinesterases; (5) a typical invertebrate acyl pocket (Sutherland et al. 1997) that contains only one conserved aromatic site F334 (290); and (6) 10 conserved aromatic amino acid residues out of 14 aromatic residues lining the catalytic gorge, present in the electric ray AChE, were also present in *N. lugens* AChE (i.e. W108, W151, Y167, W275, W326, F334, Y374, F375, Y378, and W477), in *N. cincticeps* (i.e. W144, W187, Y203, W311, W362, F370, Y410, F411, Y414, and W513), and *A. gossypii* (i.e. W123, W163, Y179, W287, W338, F346, Y386, F387, Y390, and W491), but four are not aromatic in *N. lugens* AChE: glutamic acid 94 (tyrosine 70), methionine 158 (tyrosine 121), serine 336 (phenylalanine 292), and aspartic acid 489 (tyrosine 442).

**Continued.**
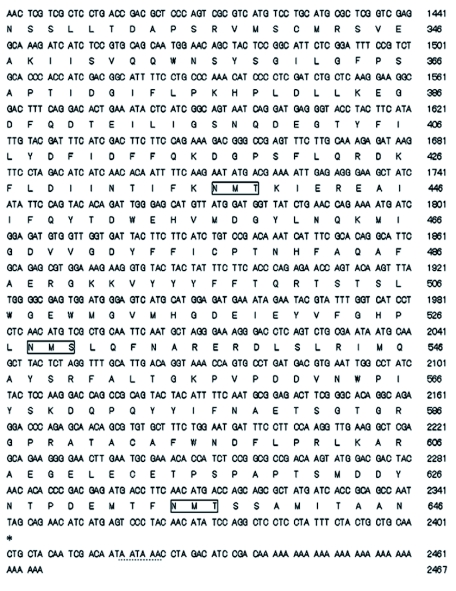

**Figure 3.**
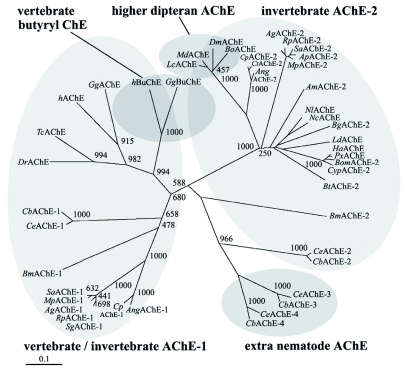
Unrooted distance neighbor-joining tree showing the phylogenetic relationships of vertebrate and invertebrate AChEs and vertebrate BuChEs. The bootstrap values with 1000 trials are indicated on branches. Scale bar indicates a distance of 0.1 amino acid substitutions per position in the sequence. Sequences are named with respect to species (abbreviated). The proteins or translated sequences with accessions are: *Nl*AChE, *Nilaparvata lugens* (AJ852420); *Ang*AChE-1, *Anopheles gambiae* (BN000066); *Cp*AChE-1, *Culex pipiens* (CAD56155); *Ag*AChE-1, *Aphis gossypii* (AAM94376); *Sg*AChE-1, *Schizaphis graminum* (AF321574); *Mp*AChE-1, *Myzus persicae* (AY147797); *Bm*AChE-1, *Boophilus microplus* (AJ223965); *Sa*AChE-1, *Sitobion avenae* (AY819704); *Rp*AChE-1, *Rhopalosiphum padi* (AY667435); *Ce*AChE-1, *Caenorhabditis elegans* (P38433); *Ce*AChE-2, *C. elegans* (061371); *Ce*AChE-3, *C. elegans* (061459); *Ce*AChE-4, *C. elegans* (061372); *Cb*AChE-I, *Caenorhabditis briggsae* (Q27459); *Cb*AChE-2, *C. briggsae* (061378); *Cb*AChE-3, *C. briggsae* (Q9NDG9); *Cb*AChE-4, *C. briggsae* (Q9NDG8); *Ha*AChE, *Helicoverpa armigera* (AAN37403); *Px*AChE, *Plutella xylostella* (AAL33820); *Ld*AChE, *Leptinotarsa decemlineata* (Q27677); *Nc*AChE, *Nephotettix cincticeps* (AF145235); *Md*AChE, *Musca domestica* (AF281161); *Lc*AChE, *Lucilia cuprina* (AAC02779); *Dm*AChE, *Drosophila melanogaster* (CG17907); *Bo*AChE, *Bactrocera oleae* (AF452052); *Am*AChE-2, *Apis mellifera* (AAG43568); *Ag*AChE-2, *Aphis gossypii* (AAM94375); *Bm*AChE-2, *Boophilus microplus* (AF067771); *Mp*AChE-2, *Myzus persicae* (AF287291); *Bom*AChE-2, *Bombyx mori* (NP_001108113); *Sa*AChE-2, *Sitobion avenae* (AY707319); *Rp*AChE-2, *Rhopalosiphum padi* (AY707318); *Ang*AChE-2, *Anopheles gambiae* (BN000067); *Cp*AChE-2, *Culex pipiens* (CAJ43752); *Ct*AChE-2, *Culex tritaeniorhynchus* (BAD06209); *Ap*AChE-2, *Acyrthosiphon pisum* (XP_001948988); *Cyp*AChE-2, *Cydia pomonella* (ABB76665); *Bt*AChE-2, *Bemisia tabaci* (ABV45414); *Bg*AChE-2, *Blattella germanica* (ABB89947); *Dr*AChE, *Danio rerio* (NM_131846); *Tc*AChE, *Torpedo californica* (X03439); *h*AChE, *Homo sapiens* (M55040); *Gg*AChE *Gallus gallus* (U03472); *h*BuChE, *Homo sapiens* (AAA99296); and *Gg*BuChE, *Gallus gallus* (AJ306928). High quality figures are available online.

Homology analysis of amino acid sequences revealed that the cDNA-deduced *N. lugens* AChE has 83% amino acid identity with that of *N. cincticeps* (accession number: AF145235-1), 78% with *L. decemlineata* (Q27677), 74% with *B. germanica* (ABB89947), 73% with *Helicoverpa armigera* (AAN37403), 72% with *Plutella xylostella* (AAL33820), 70% with *Bombyx mori* (NP_001108113), 68% with *Cydia pomonella* (ABB76665). The relationship of the predicted AChE with 44 AChEs from various species was analyzed. Phylogenetic tree indicated that the 45 AChEs assort into three lineages. *N. lugens* AChE and 21 insect AChEs formed a lineage, among which *N. lugens* AChE was most closely related to AChE of *N. cincticeps*, forming an independent cluster, suggesting that they may share the same ancestor. In this lineage, a gene loss occurred in the higher Diptera, which have lost their AChE-1 version (Russell et al. 2004). Four extra nematode AChEs (-3 and -4) from *Caenorhabditis elegans* and *Caenorhabditis briggsae* form another independent cluster with bootstrap value 966. While vertebrate and invertebrate AChEs or AChE-1s belong to the same lineage containing each copy of human and chicken butyrylcholinesterases (BuChEs). The structure of the phylogenetic tree suggests that major diversifications occurred among vertebrates and invertebrates during the evolution of this enzyme.

### Southern and Northern blot analyses

**Figure 4.**
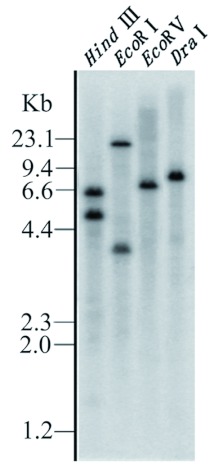
Southern blot analysis of *Nilapavarta lugens* genomic DNA. Fifteen !g of genomic DNA was digested with each of four restriction enzymes, *Eco*RI, *Eco*RV, *Hind* III and *dra*I and separated on 1.5% agarose gel. The blot was hybridized with the probe labeled by random primer using α-[^32^P] dCTP. Sizes of DNA marker (Lamda DNA/*Hin*d III) are indicated on the left. High quality figures are available online.

The cloned AChE cDNA contains one *Hind* III internal restriction site in the coding region (2040–2045), and the blot showed strong hybridization signals to approximately 5.0-and 6.0-kb fragments, suggesting there are no other *Hind* III sites in the internal sequence of this cDNA. However, the cDNA does not contain any restriction sites for the enzymes *Eco*RI, *Eco*RV and *DraI*. In *Eco*RV and *Dra*I digested DNA, strong hybridization was seen to 7.0- and 8.5-kb fragments, respectively. When the probe was used to hybridize to the *Eco*RI digested fragments, the blot showed strong hybridization to approximately 16- and 3.8-kb fragments (Figure 4). The additional hybridization fragment in the blot of *Eco*RI digested DNA can be explained by the presence of *Eco*RI sites in the introns of AChE cDNA. There are nine introns in the AChE gene of *D. melanogaster* (Fournier et al. 1989). In this study, the additional hybridization fragment in the blot of *Eco*RI-digested DNA may be explained by the probe corresponding to the region containing most of the exons of AChE gene in *D. melanogaster,* therefore, multiple bands would be expected. Southern blot analysis suggested that there is likely a single copy of this AChE gene in *N. lugens.*

**Figure 5.**
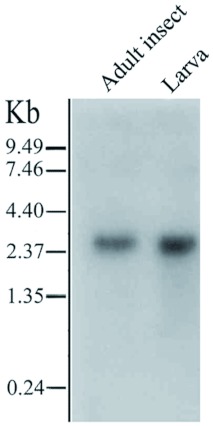
Northern blot analysis of poly(A)^+^ RNA purified from *Nilapavarta lugens* mRNA (3 !g per lane) was separated on 1.5% denaturing, formaldehyde agarose gel. The blot was hybridized with the probe labeled by random primer using α-[^32^P] dCTP. Sizes of RNA marker (GIBCO / BRL) are indicated on the left. High quality figures are available online.

This gene exhibited a very low level of mRNA expression because no hybridization signal could be detected in Northern blot analysis using total RNA. When the poly(A)^+^ RNA was used for Northern blot analysis, a distinctive 2.6-kb transcript was revealed in adult insects and larvae (Figure 5). This transcript size matches well with the 2.5-kb of *N. lugens* AChE cDNA.

### Detection of the mutations in acetylcholinesterase from resistant strain

An inner AChE2 cDNA fragment with 2065 bp (base position 320–2384 in the full cDNA sequence) containing the complete coding sequence was amplified from the 10 individual larvae. The 10 fragments were analyzed for genotype by direct sequencing. The result showed that the cDNA sequences were homozygous, and no genetic polymorphisms were observed within the same strain individuals. But there were three base substitutions in the resistant strain cDNA (accession FM866396), compared with the susceptible strain. Two of the three bases were in the same codon and resulted in a nonsynonymous amino acid replacement (GGG by AGC, base position 856–858, resulting in Gly185Ser (Gly118 in *T. californica*)). The other substituted base is located at the position 2134 and is a synonymous alteration, resulting in change of the codon TTC to TTT, both of which code for Phe. Thus this bp change is likely irrelevant to the resistance.

## Discussion

In this study, a full cDNA encoding AChE was isolated from *N. lugens* and characterized in detail. The *N. lugens* AChE has the highest amino acid identity (83%) with that from *N. cincticeps.* Phylogenetic analysis also revealed that *N. lugens* AChE was most closely related to *N. cincticeps,* and it belongs to AChE2 subgroup (Figure 3). The deduced *N. lugens* AChE has the major conserved features found in AChE of *T. californica* (Sussman et al. 1991). Fourteen aromatic residues in *T. californica* form the gorge, which directs acetylcholine to the active site serine, and most of these residues are present in AChE from other organisms studied. In this work, four out of the 14 residues were not conserved aromatic in the deduced AChE from *N. lugens* (Figure 2), and the other thirteen insects were found to lack the four aromatic residues as well, suggesting that whether the four residues are conservative will not affect the enzyme activity in some insects. There were four predicted N-glycosylation sites in the mature AChE in *N. lugens*. The four sites are likely to be necessary for the function of this enzyme, perhaps for glycophospholipid attachment, which is the main form of membrane attachment of AChEs in invertebrates (Gagney et al. 1987).

It has been known that vertebrates and invertebrates have different forms of *AChE* genes (Grauso et al. 1998). Vertebrates have a variety of AChE forms encoded by a single gene. These forms differ in the number of subunits and the way they are linked to cell membranes. These AChEs contain the same catalytic domain and catalytic activity but are translated from different mRNAs generated by alternative splicing of the single gene (Li et al. 1991). Invertebrates also have different forms of AChE; some nematodes have different AChEs encoded by more than one gene, and the different forms of AChE have different catalytic activities (Gao et al. 2002). Previous studies revealed that some insects have two different AChEs, which are either orthologous or paralogous to *Drosophila Ace* (Li and Han 2002; Nabeshima et al. 2003, 2004; Chen and Han 2006; Shang et al. 2007; Badiou et al. 2007). Actually, four gene duplication events and at least one gene deletion event occurred in the evolution of AChEs from nematodes to humans (Russell et al. 2004). The loss of the AChE-1 gene took place in insects, specifically in the higher Diptera. Because AChE-1 processes acetylcholine in the majority of insect and arthropods, the higher Diptera without AChE-1 must rely on their single AChE enzyme (derived from the ancestral ace-2) to execute these functions (Harel et al. 2000; Weill et al. 2002). In this study, Southern and Northern blot analyses revealed that there is probably one copy of the AChE gene in the *N. lugens* genome and one AChE transcript in the transcriptome. However, these data are too limited to draw a conclusion that there is truly a single AChE gene in *N. lugens.* Whether there is a second gene encoding AChE paralogous to *Drosophila* AChE is an interesting question currently under exploration.

Many agricultural and medical pests have developed resistance to insecticides by decreasing the sensitivity of AChE. Specific amino acid substitutions at several positions in AChE were shown to cause a decreased sensitivity of AChEs to insecticides in some insects, pointing to the importance of AChE protein primary structure in insecticide resistance (Li and Han 2004; Nabeshima et al. 2003, 2004; Hsu et al. 2006, 2008; Alout et al. 2007; Kakani et al. 2008; Magaña et al. 2008). The absence of protein polymorphism attributable to insecticide insensitivity was reported in *N. cincticeps* (Tomita et al. 2000). Independent duplications of the AChE gene confer insecticide resistance in the mosquito *Culex pipiens* (Labbé et al. 2007). In this work, the altered AChE predicted from the cDNA cloned from the resistant *N. lugens* contained an amino acid replacement, Gly185Ser. Gly185 (Gly118) is an important residue that forms the oxyanion hole with Ala273 (Ala201) and Gly186 (Gly119) in the active site of AChE. The oxyanion hole formed by the peptidic NH group from these three residues forms hydrogen bonds with the carbonyl oxygen of the substrate or inhibitor and performs the function of stabilizing the negative charge on the anionic moiety of the ligand (Zhang et al. 2002). A mutation at this site is likely to change the affinity of AChE for its substrates and inhibitors. A previous study revealed that Gly221Ser in *A. gossypii* (Gly119) in AP-AChE from OP resistant *Cx. pipiens* and *Anopheles gambiae* mosquitoes was a replacement of another of the three residues forming the oxyanion hole (Weill et al. 2003). Subsequently, the third amino acid Ala302 (Ala201) of the three residues was found to be replaced by a Ser in *A. gossypii* AChE, resulting in reduced susceptibility of the H-16 strain to two organophosphorous insecticides, fenitrothion and malathion (Toda et al. 2004). The resistant *N. lugens* strain that was studied here exhibited a 25-fold decrease in methamidopho sensitivity. The replacement, Gly185Ser in the altered AChE, likely confers this insecticide insensitivity.

People once controlled *N. lugens* by using insecticides, but this strategy does not work effectively today. Continuous use of insecticides has reduced the biological regulatory function of natural enemies, resulting in resurgence and insecticideresistance in this pest (Sujatha and Regupathy 2003). To minimize these problems, it is necessary to reduce the usage amount of insecticides and to find a more environmentally-friendly control strategy. The cloned AChE cDNA in this study revealed the molecular properties of the AChE from *N. lugens*. The altered AChE with the amino acid substitution (Gly185Ser) might confer methamidopho insensitivity in the resistant strain. Further investigation is needed to elucidate the mechanism of this mutation in resistant *N. lugens*, which would be expected to help in the effective control and resistance management of this pest.

## Abbreviations

AChE: acetylcholinesterase;
GSP: gene specific primer
